# Pressure Effects on the Magnetic Phase Diagram of the Ce*NM*Sb_2_ (*NM*: Au and Ag): A DFT Study

**DOI:** 10.3390/ma13102237

**Published:** 2020-05-13

**Authors:** Mowafaq Mohammad Alsardia, Jaekyung Jang, Joo Yull Rhee

**Affiliations:** Department of Physics, Sungkyunkwan University, Suwon 16419, Korea; mowafaq@g.skku.edu (M.M.A.); jkjang0408@gmail.com (J.J.)

**Keywords:** electronic structure, heavy fermion, spin-orbit interaction, magnetic phase, magnetic easy axis

## Abstract

We explore the influence of pressure on the magnetic ground state of the heavy-fermion antiferromagnet (ferromagnet) CeAuSb${}_{2}$ (CeAgSb${}_{2}$) using first-principles calculations. The total-energy differences obtained by including the spin-orbit interactions and the on-site Coulomb potential for the Ce-derived 4*f*-orbitals are necessary to realize the accurate magnetic ground state of Ce*NM*Sb${}_{2}$ (*NM*: Au and Ag). According to our results, the appearance of a new magnetic phase of CeAuSb${}_{2}$ (CeAgSb${}_{2}$) at the pressure of 2.1 GPa (3.5 GPa) is due to the rotation of the magnetic easy axis from the <001> to the <100> direction. Additionally, our data confirm that CeAgSb${}_{2}$ is antiferromagnetic (AFM) above a critical pressure $P_{c}$, and such a tendency is expected for CeAuSb${}_{2}$ and remains to be seen. Through the spin-orbit-coupling Hamiltonian and detailed information on the occupation of individual 4*f*-orbitals of the Ce atom obtained by the electronic-structure calculations, we can deduce the rotation of the magnetic easy axis upon the application of pressure. According to the present and previous studies, the differences among the magnetic properties of Ce*NM*Sb${}_{2}$ (*NM*: Cu, Ag and Au) compounds are not due to the different noble metals, but due to the subtle differences in the relative position of Ce atoms and, in turn, different occupations of Ce 4*f*-orbitals.

## 1. Introduction

In recent years, considerable interest has been devoted to examining Ce-based compounds as they display numerous intriguing physical properties such as heavy-fermion behavior, strong magnetic anisotropy, a field-tuned quantum critical point (QCP), unusual transport and thermodynamic properties, and unconventional superconducting states [1,2,3,4,5,6,7]. Hence, such unique physical properties may help in paving the way for important technological applications in magnetic-storage technology and spin-electronics devices and affords the possibility to design materials, e.g., exploiting superconductivity or thermoelectrics. These compounds result in an alternative and interesting approach to investigate their physical properties in the vicinity of QCP [1,2,3,6,8,9,10,11,12,13,14]. In this class of compounds, it was suggested that the competition between the Ruderman–Kittel–Kasuya–Yosida (RKKY) interaction and the Kondo effect played an important role in the appearance of different phases [15,16,17]. Since both are the consequences of the hybridization between the conduction electrons and the *f*-electrons, the competition can be controlled by external stimuli, which can modulate the strength of the hybridization. The external stimuli are the magnetic field, chemical composition, pressure, and so on. Eventually, one can have the opportunity to control the magnetic transition temperature to *T* = 0 K [6,7,18] through those external stimuli. Once the strengths of RKKY interaction and the Kondo effect become comparable, quantum critical behavior may take place, and a superconducting state could be formed close to the critical point.

The application of the hydrostatic pressure to Ce-based compounds continuously changes the strength of the magnetic exchange interaction $J_{\mathrm{cf}}$ because of the variation of interatomic distances and the oscillating nature of the RKKY interaction. With increasing $J_{\mathrm{cf}}$, the magnetic moments of the localized Ce *f*-electrons are screened by the spin polarization of the conduction electrons, inducing the spin fluctuations and a reduced magnetic moment and transition temperature. At the QCP where the magnetic order is completely suppressed, a non-Fermi-liquid character and/or unconventional superconductivity are often observed. A nonmagnetic Fermi-liquid state with strong spin fluctuations is established for a large $J_{\mathrm{cf}}$ beyond the critical value of $J_{c}$ at a QCP. Consequently, the aforementioned aspects in such a class of compounds make the magnetic phase diagram extremely complicated, and the nature of the pressurized state depends qualitatively on a delicate balance between the two competing low-energy scales and, in particular, on the proximity of the specific system to the QCP.

CeAuSb${}_{2}$ demonstrates a complex magnetic phase diagram under pressure [8], probably reflecting the interplay of the pressure dependence of the RKKY, Kondo, and crystal-electric-field (CEF) effects and suggesting the possibility of a magnetic QCP. Applying pressure suppresses the magnetic transition temperature at first; however, new magnetic states have been realized with increased pressure above 2.1 GPa. The pressure-induced new magnetic phase adopts a dome shape, which is accompanied by an additional phase when the hydrostatic pressure exceeds 4.7 GPa. Moreover, Seo et al. found evidence in the temperature-magnetic field phase diagrams of CeAuSb${}_{2}$ under applied pressure that the magnetic order of CeAuSb${}_{2}$ is associated with a structural transformation to a superstructure, which is probably due to the lowering of the symmetry of the low-temperature phase of CeAuSb${}_{2}$, which is in the P4/nmm space group at a high temperature, thus signaling nematic order [19]. In CeAgSb${}_{2}$, the ferromagnetism is suppressed at a critical pressure $P_{c}$ = 3.5 GPa, and a new magnetic phase (possibly antiferromagnetic) emerges above 2.7 GPa with a maximum value of $T_{N}$∼ 6 K at 4.4 GPa before being suppressed at ∼5.0 GPa [9]. However, Logg et al. [20] argued that the magnetic order in CeAgSb${}_{2}$ is very sensitive to hydrostatic pressure, which enhances the Kondo screening of the 4$f^{1}$ site, and above 3.5 GPa, the initial magnetic order completely disappears. Moreover, the observed magnetic phase was not traced in magnetization measurements under pressure. The exact nature of the pressure-dependent magnetic and electronic properties remains unclear and not fully understood in spite of intensive experimental efforts. Therefore, one must pay deliberate attention to the characteristics of the spin and orbital in *f*-electron systems since the strong spin-orbit interactions keep them tightly coupled with each other. Although most of the advancements in this field of research have been achieved owing to advances in experimental techniques [8,21,22,23,24], theoretical studies are highly desirable for providing better and systematic understanding.

Furthermore, in our previous work [25,26], we investigated the electronic structures of antiferromagnetic Ce*NM*Sb${}_{2}$ (*NM*: Cu and Au), as well as the ferromagnetic CeAgSb${}_{2}$ compound to elucidate the role of 4*f* electrons in determining the ground state. We concluded that the effects of spin-orbit coupling (SOC) of Ce 4*f* electrons and their orbital occupancy, as well as the on-site Coulomb interaction *U* of Ce played a fundamental role in specifying the magnetic easy axes. Moreover, the two Ce atoms in a unit cell of ferromagnetic (FM) CeAgSb${}_{2}$ compound should be treated inequivalently, similar to the antiferromagnetic (AFM) sister compound CeAuSb${}_{2}$ [26]. As part of an effort to better understand the anisotropic magnetic and electronic behavior of the Ce-based heavy-fermion family, the Ce*NM*Sb${}_{2}$ (*NM*: Au and Ag) compounds were chosen, making them a logical candidate to investigate. The Ce*NM*Sb${}_{2}$ compounds crystallize in the ZrCuSi${}_{2}$-type tetragonal structure where the noble-metal layer is contained between two CeSb layers [21,23,27,28,29,30,31]. At ambient pressure, the CeAuSb${}_{2}$ (CeAgSb${}_{2}$) orders antiferromagnetically (ferromagnetically) between 5 and 6.8 K (below 9.6 K) with an easy axis along the <001> direction [8,9,22,27].

Herein, we report the theoretical investigation of the pressure-dependent magnetic and electronic properties of Ce*NM*Sb${}_{2}$ compounds by means of the total-energy difference between two different orientations of the magnetization, orbital occupancy, and the SOC energy of *f*-orbitals obtained by a similar manner to our previous work. We found that the relative positions of Ce atoms played a more fundamental role in the magnetic properties of Ce*NM*Sb${}_{2}$ compounds than the different noble metals.

## 2. Calculation Details

We utilized the WIEN2k code [32] for the electronic-structure calculations, which was implemented with the full-potential (linearized-)augmented-plane-wave plus local orbital method. The exchange-correlation functional is the generalized-gradient approximation in the form proposed by Perdew, Burke, and Ernzerhof [33]. The muffin-tin radii were 2.5 a.u. for all atoms. The value for the convergence parameter was taken to be $RK_{\max}$ = 7 (resulting in 120 and 88 augmented plane waves for the basis functions, for CeAuSb${}_{2}$ and CeAgSb${}_{2}$, respectively. We varied the unit-cell volume and c/a ratio utilizing the 2D optimization feature of the WIEN2k package. Since this study dealt with less than a 1.0 mRy difference in the total energy, a mesh of ∼1204 k-points was considered in the irreducible wedge of the first Brillouin zone, which corresponded to the grids of 23×23×9 in the Monkhorst–Pack scheme [34] for precise total-energy calculations. We performed AFM/FM spin-polarized (SP) calculations. The orbital-dependent potentials, the so-called GGA+*U* method [35], and SOC interactions were included. Commonly, the *U* value for the 4*f* Ce atom is utilized from 5 eV to 7 eV in most cases. Thus, we set the on-site Coulomb interaction to be *U*∼ 6 eV (CeAuSb${}_{2}$) and 7 eV (CeAgSb${}_{2}$) and the exchange parameter *J* = 0 for the effective potential $U_{\mathrm{eff}}$ = U−J. The experimental lattice parameters used in the present calculations for CeAuSb${}_{2}$ and CeAgSb${}_{2}$ were taken from [27,36], respectively. We utilized the SOC Hamiltonian as expressed in [25], for $E_{\mathrm{SOC}}$ calculation. We considered two different directions of magnetizations such as <001> and <100>. Two different magnetic phases, FM and AFM, were used to compare the total energies for CeAgSb${}_{2}$. The unit cell of the investigated compounds contained two formula units.

## 3. Results and Discussion

**Figure 1:**
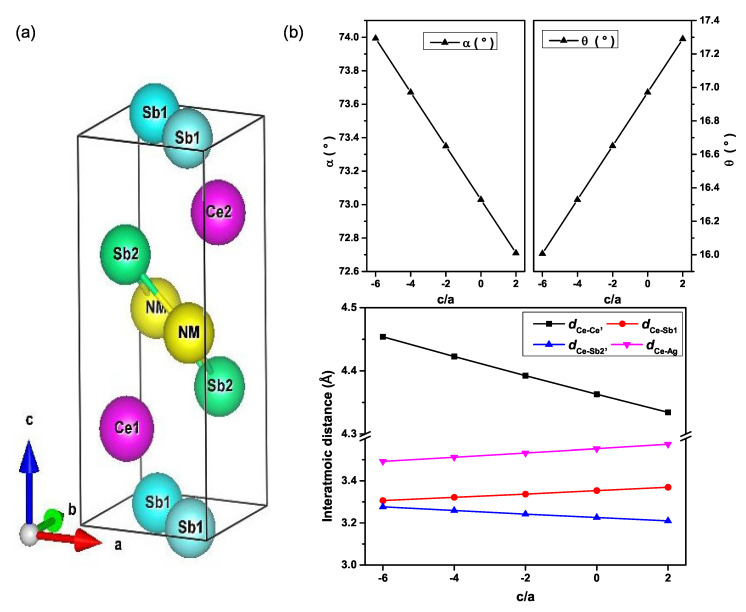
(**a**) Crystal structure of Ce*NM*Sb${}_{2}$ and (**b**) variations of crystallographic data owing to the change of the c/a ratio at constant volume for the CeAgSb${}_{2}$ compound.

In order to understand the effect of the pressure on the magnetic properties of Ce*NM*Sb${}_{2}$, we carried out first-principles calculations with different magnetization orientations, one with the magnetization along the <001> direction and the other along the <100> direction, due to the lack of information about the magnetic easy axis of the new magnetic phases above $P_{c}$ in both compounds. The total energies ($E_{\mathrm{tot}}$) were computed by varying the volume and c/a ratio of the unit cell. An analysis and overview of the volume and c/a ratio of the unit cell variation process effects on the crystallography environment are presented in Figure 1. The Sb-Ce-Sb bond angle (α), $d_{\mathrm{Ce}-\mathrm{Ce}}$ (second-nearest neighbor), and $d_{\mathrm{Ce}-\mathrm{Sb}2}$ were linearly decreasing. The tilt angle of the Ce-Sb bond (θ) with respect to the *ab*-plane, as well as $d_{\mathrm{Ce}-\mathrm{Sb}1}$ and $d_{\mathrm{Ce}-\mathrm{Ag}}$ were found to increase linearly with increasing the c/a ratio. Here, $d_{A-B}$ stands for the distance between Atom A and Atom B. One must note that there were two crystallographically different Sb atoms as presented in Figure 1. The crystallography data reported here revealed the sensitivity of the Ce atoms’ relative position to the pressure resulting from varying the c/a ratio through the 2D optimization. Consequently, we argued that a subtle difference in the relative position of Ce atom led to different occupations of Ce 4*f*-orbitals, resulting in different magnetic easy axes, as presented in the rest of this paper.

**Figure 2:**
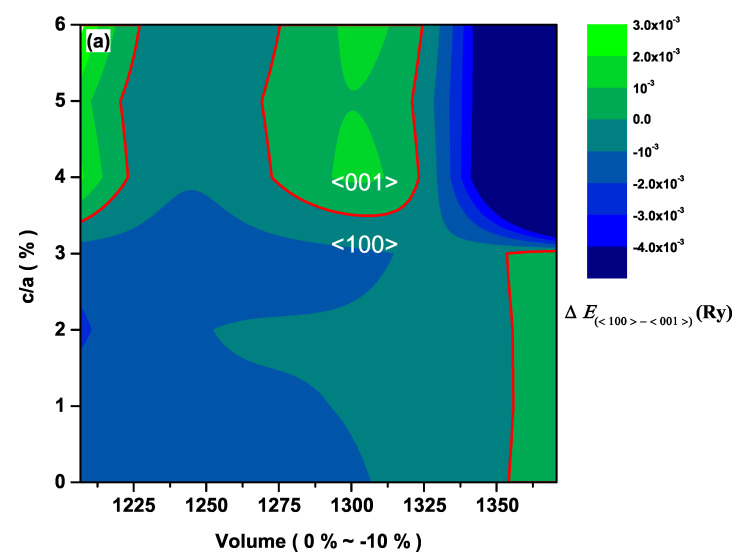
Total-energy differences, $\Delta E_{\mathrm{tot}}=E_{\mathrm{tot}}^{100}-E_{\mathrm{tot}}^{001}$, for the CeAuSb${}_{2}$ compound. The thick red curve corresponds to $\Delta E_{\mathrm{tot}}=0$, i.e., the phase boundary between the cases with the magnetization along the <001> and <100> directions.

**Figure 3:**
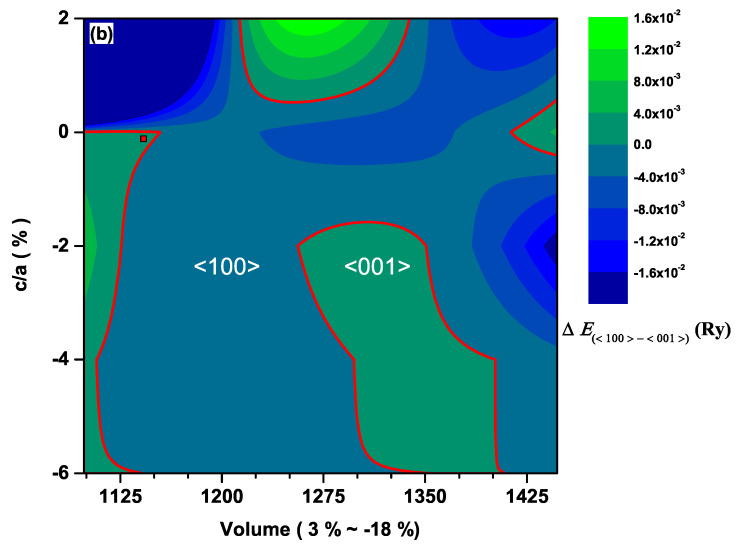
The same as Figure 2 except for the CeAgSb${}_{2}$ compound. The red square symbol corresponds to the $E_{\mathrm{SOC}}$ calculated above $P_{c}$.

The contour plot of the total energy difference, $\Delta E_{\mathrm{tot}}=E_{\mathrm{tot}}^{100}-E_{\mathrm{tot}}^{001}$ between the cases with the magnetization along the <001> and <100> directions for CeAuSb${}_{2}$ and CeAgSb${}_{2}$, are displayed in Figure 2 and Figure 3, respectively. If $\Delta E_{\mathrm{tot}}>0$, the <001> direction is the easy axis, and the <100> direction is the easy axis otherwise. We have to keep in mind that the energy differences between the cases for $M_{<001>}$ and $M_{<100>}$ in such a class of compounds are in order of mRy. As shown in both Figure 2 and Figure 3, it is obvious that there were some regions where $\Delta E_{\mathrm{tot}}<0$, implying that the <100> direction could be the easy axis when an appropriate pressure is applied. Hence, it was unambiguous that the pressure could cause a rotation of the easy axis.

**Figure 4:**
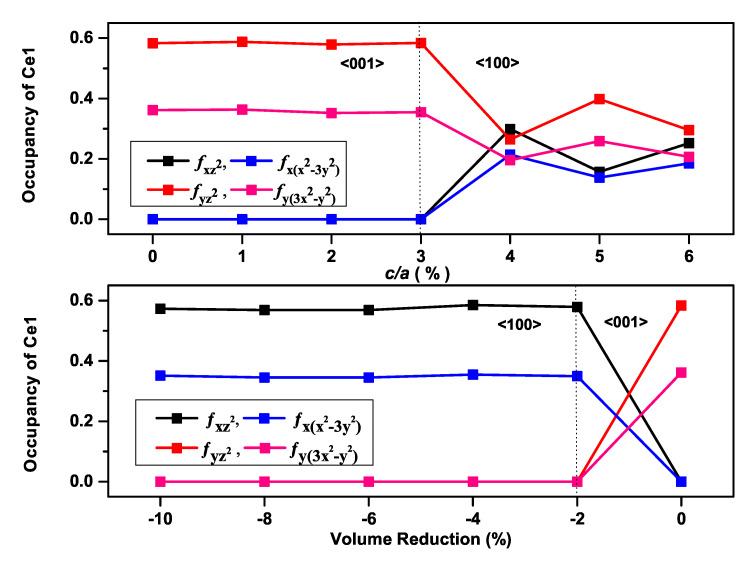
Occupancies of different 4*f* orbital states’ number plots of the Ce atom with the magnetization along <001> for the CeAuSb${}_{2}$ compound at constant volume (top) and a constant c/a ratio (bottom).

**Figure 5:**
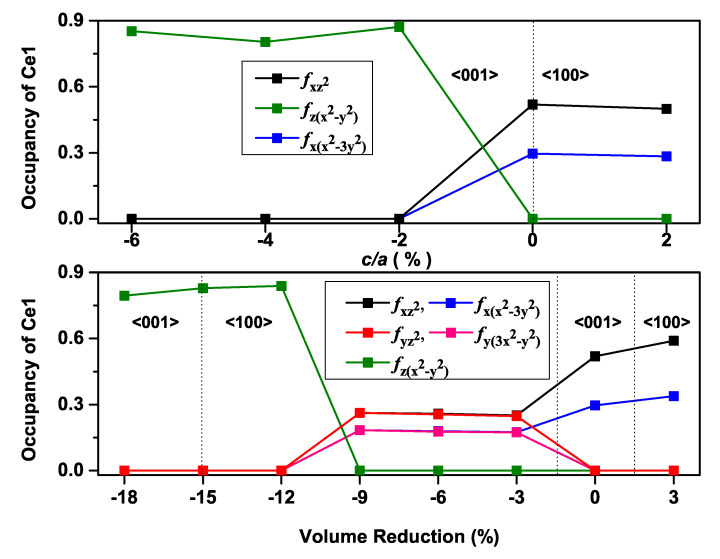
The same as Figure 4 except for the CeAgSb${}_{2}$ compound.

According to our previous findings on AFM Ce*NM*Sb${}_{2}$ (*NM*: Cu and Au) compounds [25,26], the eigenvectors of occupied 4*f*-orbitals along the easy axis were only those with $m_{l}=\pm$2, for CeCuSb${}_{2}$, leading to an easy axis on the *ab*-plane, and those with $m_{l}=\pm$ 1, ±3, for CeAuSb${}_{2}$, with an out-of-plane easy axis, which was ascribed to the different relative positions of Ce atoms rather than the difference of the SOC effects of the noble metals. Additionally, for the accurate prediction for the magnetization easy axis of AFM Ce*NM*Sb${}_{2}$ compounds, it was required to include both SOC and the on-site Coulomb interaction *U* of the Ce atom in the spin-polarized calculation for the accurate prediction of the magnetization easy axis [25]. Furthermore, uniform density-of-state (DOS) plots were obtained for valence electrons with different magnetization directions. Only Ce 4*f*-bands displayed an obvious difference. We picked one point as an example to present the partial-DOS (PDOS), which defined the number of electron states per unit energy range on a particular atomic orbital to elucidate the effect of varying the c/a and the unit cell volume on the 4*f*-orbital occupations for the Ce atom with the magnetization along <001>. It is obvious from Figure 4 that different occupations occurred when the fluctuation took place from the easy axis to the hard axis as the c/a ratio (volume) increased (decreased) for the CeAuSb${}_{2}$ compound, and this was likewise true for the CeAgSb${}_{2}$ compound as displayed in Figure 5, which reflected the sensitivity of the Ce atom occupancy to the exerted pressure.

Therefore, on the way to understanding the 4*f* electrons more systematically under the pressure exerted by varying the volume and c/a ratio of the unit cell, we analyzed the orbital characteristics of occupied Ce 4*f* bands.

**Figure 6:**
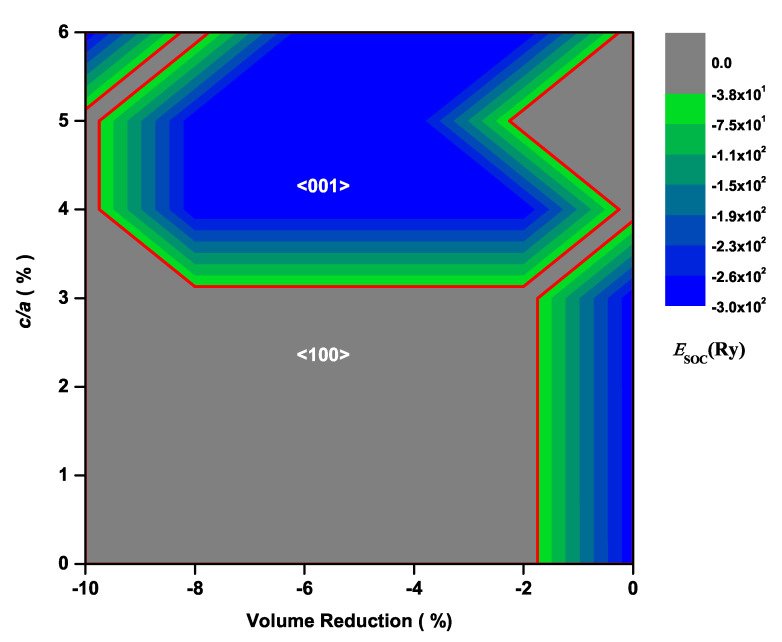
$E_{\mathrm{SOC}}$ for the CeAuSb${}_{2}$ compound. The thick red curve corresponds to $E_{\mathrm{SOC}}=0$, i.e., the phase boundary between the easy axis along the <100> and <001> directions.

We speculated that the variation of the relative positions of Ce atoms played an important role in the different orbital occupation, this different orbital occupation resulting in different $E_{\mathrm{SOC}}$, and consequently, the change in the relative position of Ce atoms produced the rotation of the magnetic easy axis. To verify this speculation, we examined SOC matrices for 4*f*-orbitals through the volume and c/a ratio of the unit cell variations by following the same reasoning as in our previous work on AFM Ce*NM*Sb${}_{2}$ compounds [25,26]. Since we knew that the sign of $E_{\mathrm{SOC}}$ was a key reference to judge the magnetization easy axis and the $M_{<100>}$ matrix elements gave null results [25], we simply considered $M_{<001>}$ and calculated $E_{\mathrm{SOC}}$ along <001> where the negative sign of $E_{\mathrm{SOC}}$ must be obtained if the <001> direction was the easy axis. As shown in Figure 6, it was evident that there were some regions corresponding to $\Delta E_{\mathrm{tot}}>0$ where $E_{\mathrm{SOC}}<0$, implying that the energy gain of CeAuSb${}_{2}$ due to the SOC was negative. That led to the state with magnetization along the <001> direction being energetically higher than that with magnetization along the <100> direction. The opposite scenario would be true when the <100> direction was the easy axis. Further, there was no need to repeat the same calculation for the CeAgSb${}_{2}$ compound [26], since the only difference between the present and the previous calculations was the number of *k*-points. Rather, we picked one point above $P_{c}$, as indicated as the red square symbol in Figure 3 and calculated $E_{\mathrm{SOC}}$. According to the calculation, $E_{\mathrm{SOC}}$ was −2.7858 $\times \;10^{2}<0$. This implied that the magnetic easy axis was along the <001> direction. Therefore, different relative positions of Ce atoms, equivalently different occupations of 4*f*-orbitals of Ce in these compounds, led to the different orientations of the magnetic easy axes.

**Figure 7:**
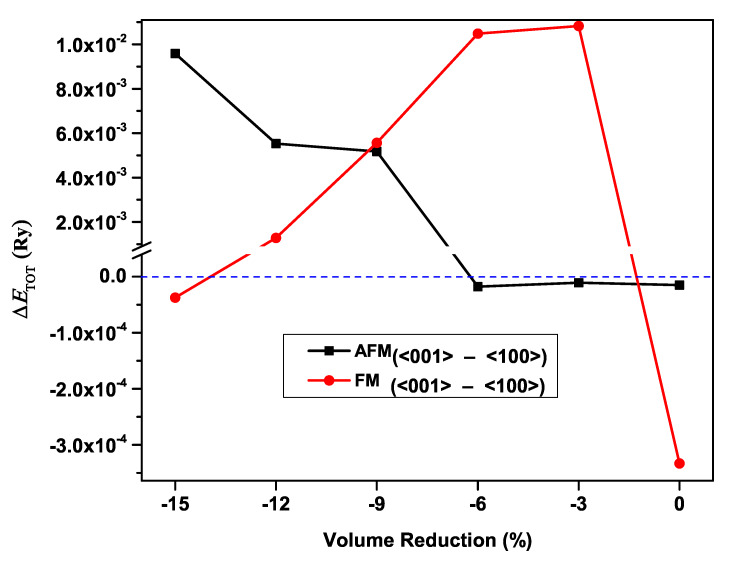
Pressure dependence of the ferromagnetic (FM) and antiferromagnetic (AFM) states of the CeAgSb${}_{2}$ compound with different orientations of the magnetization, one with the magnetization along the <001> direction and the other along the <100> direction.

In an effort to clarify how the magnetic properties of CeAgSb${}_{2}$ were affected under applied hydrostatic pressure, considerable experimental investigation was performed [9,20]. Yet, there was a need to verify the response of the magnetic properties of CeAgSb${}_{2}$ at various pressures. For that purpose, we carried out the detailed investigation of the effects of pressure considering the FM and AFM coupling between the moments of the two Ce atoms. For each configuration, the energy difference between the $\Delta E_{\mathrm{TOT}<001>-<100>}^{\mathrm{FM}}$ and $\Delta E_{\mathrm{TOT}<001>-<100>}^{\mathrm{AFM}}$ states of CeAgSb${}_{2}$ are presented in Figure 7. If $\Delta E_{\mathrm{TOT}}$ was negative, the <001> direction was the easy axis, and the <100> direction was the easy axis otherwise. As shown in Figure 7, it was obvious that there were some regions where $\Delta E_{\mathrm{TOT}}^{\mathrm{AFM}}<\Delta E_{\mathrm{TOT}}^{\mathrm{FM}}$, implying that the AFM state was energetically favorable. If ΔE was nearly zero, it meant that paramagnetism was the ground state. Consequently, it was evident that the pressure may result in the rotation of the easy axis, as well as the magnetic phase. Accordingly, we agreed that the appearance of a new magnetic phase of CeAgSb${}_{2}$ at a pressure of 3.5 GPa was AFM. In our previous study [25,26], we found that the SOC of the Ce 4*f* electron played a rather important role in the determination of the magnetic easy axis of Ce*NM*Sb${}_{2}$ (*NM*:Cu, Au and Ag) compounds under ambient pressure. This result was opposite our intuition. It is natural, at first glance, to consider the difference of the SOC of noble metals to be the key factor to determine the magnetic easy axis of these compounds because they crystallize in the same crystallographic structure and are isoelectronic. The only electronic differences were the number of core electrons, which caused different SOC, of noble metals. Not only our previous study, but also the present study indicated that the subtle difference in the relative positions of Ce atoms in different compounds was the key factor to determine the magnetic easy axis. The subtle difference in the relative positions of Ce atoms led to different occupancies of the Ce 4*f*-orbital, which determined the sign of $E_{\mathrm{SOC}}$. Therefore, we could argue that the difference in the relative position of Ce atoms played a more fundamental role in the determination of the magnetic easy axis of these compounds than the difference in noble metals.

## 4. Summary

The electronic structures’ calculations were done using the so-called GGA+*U* method to obtain the total energies, PDOSs, and $E_{\mathrm{SOC}}$’s of the tetragonal Ce*NM*Sb${}_{2}$ (*NM*: Au and Ag) compound. Our calculations confirmed that the correct ground state of these compounds could be obtained only by including the spin-orbit interactions and the on-site Coulomb potential for the Ce-derived 4*f*-orbitals. Through total-energy difference plots, we found that the variation of the pressure could cause a rotation of the easy axis. A different magnetic easy axes was led by the different occupations of Ce 4*f*-orbitals due to a subtle difference in the Ce relative positions. The pressure-dependent magnetic phase diagram of the Ce*NM*Sb${}_{2}$ compound was investigated, and our data confirmed that the AFM (FM) CeAuSb${}_{2}$ (CeAgSb${}_{2}$) disappeared continuously at a pressure near 2.1 GPa (3.5 GPa) and provided evidence that CeAgSb${}_{2}$ was AFM above $P_{c}$. Contrary to our simple belief, the key factor for the determination of magnetic phase of Ce*NM*Sb${}_{2}$ (*NM*: Cu, Ag and Au) was not the difference in noble metals, but the difference in the relative positions of Ce atoms.
